# Identification of Genes/Proteins Related to Submergence Tolerance by Transcriptome and Proteome Analyses in Soybean

**DOI:** 10.1038/s41598-019-50757-1

**Published:** 2019-10-11

**Authors:** Yanhui Lin, Wei Li, Yanwei Zhang, Changjian Xia, Yun Liu, Caijie Wang, Ran Xu, Lifeng Zhang

**Affiliations:** 1 Crop Research Institute, Shandong Academy of Agricultural Sciences, Jinan, China; 2 Institute of Food Crops, Hainan Academy of Agricultural Sciences, Haikou, China; 3 Haikou Cigar Research Institute, Hainan Provincial Branch of China National Tobacco Corporation, Haikou, China; 4 State Key Laboratory for Biology of Plant Diseases and Insect Pests, Institute of Plant Protection, Chinese Academy of Agricultural Sciences, Beijing, China; 5 College of Agriculture, Yangtze University, Jingzhou, China

**Keywords:** Plant molecular biology, Flooding

## Abstract

Flooding can lead to yield reduction of soybean. Therefore, identification of flooding tolerance genes has great significance in production practice. In this study, Qihuang 34, a highly-resistant variety to flooding stress, was selected for submergence treatments. Transcriptome and proteome analyses were conducted, by which twenty-two up-regulated differentially expressed genes (DEGs)/differentially expressed proteins (DEPs) associated with five KEGG pathways were isolated. The number of the DEGs/DEPs enriched in glycolysis/gluconeogenesis was the highest. Four of these genes were confirmed by RT-qPCR, suggesting that glycolysis/gluconeogenesis may be activated to generate energy for plant survival under anaerobic conditions. Thirty-eight down-regulated DEGs/DEPs associated with six KEGG pathways were identified under submergence stress. Eight DEGs/DEPs enriched in phenylpropanoid biosynthesis were assigned to peroxidase, which catalyzes the conversion of coumaryl alcohol to hydroxy-phenyl lignin in the final step of lignin biosynthesis. Three of these genes were confirmed by RT-qPCR. The decreased expression of these genes led to the inhibition of lignin biosynthesis, which may be the cause of plant softening under submergence stress for a long period of time. This study revealed a number of up-/down-regulated pathways and the corresponding DEGs/DEPs, by which, a better understanding of the mechanisms of submergence tolerance in soybean may be achieved.

## Introduction

Soybean is the most important legume crop in the world, which is rich in protein, oil and other nutrients^1^. With the continuous growth of the global population, the demand for soybean is also increasing. In recent years, climate change has shown great impacts on precipitation in many areas, which, in turn, affects the production of soybean due to its high sensitivity to flooding stress. Alternative to oxidative respiration, plants employ glycolysis to generate ATP and alcohol fermentation to produce NAD + required for sustaining the EMP pathway^2^. Flooding stress is a serious obstacle to plant growth and development, leading to yield reduction and even death^2,3^. Therefore, the study on soybean flooding tolerance has great significance in agricultural production.

Rice is one of the representative plants in flooding tolerance studies; many genes related to deep water stress have been cloned, such as *Sub1A*, *SK1* and *SK2*^4–7^. In soybean, many quantitative trait loci (QTLs) associated with flooding tolerance have been reported, such as *ft1-7* and *Sft1-4*^8–10^.

In recent years, with the rapid development of modern molecular biology and bioinformatics, plant responses to flooding have been studied by transcriptome sequencing in many plants, including *Sesbania*, *Arabidopsis thaliana*, rice, maize and soybean^11–16^. These studies provide an effective basis for exploring the mechanism of responses to flooding at the transcriptional level in crops. In rice, the response to deep-water stress has been investigated by RNA-Seq. One study showed that jasmonic acid participates in internode elongation and improves flooding tolerance^17^. Expression profiles of soybean have been performed under drought and flooding stress; the results show that many genes involved in photosynthesis, chlorophyll biosynthesis, cell wall biosynthesis and starch and glucose metabolism are affected by two extreme water stresses^18^. It has also been reported that genes related to glycolysis and alcohol fermentation, ethylene biosynthesis and pathogen defense are up-regulated under flooding stress, while genes related to metabolism are down-regulated in soybean and *Sesbania cannabina*^11,15^. Proteome studies also show that proteins involved in fermentation, removal of reactive oxygen species, glycolysis, disease resistance and defense response are affected under flooding stresses^19,20^. Many reports have been conducted on the responses to flooding stress through transcriptome or proteome in soybean. However, few studies have been reported to explore the pathways and the differentially expressed genes (DEGs)/differentially expressed proteins (DEPs) through association analysis of transcriptome and proteome under flooding stresses.

In this study, the cultivated variety Qihuang 34 was treated by submergence, and the untreated Qihuang 34 was used as a control. The transcriptome and proteome association analysis was performed, from which eleven DEGs/DEPs encoding key enzymes were enriched in the glycolysis/gluconeogenesis pathway. Glycolysis/gluconeogenesis was activated to produce ATP for plant survival under anaerobic conditions. Eight down-regulated DEGs/DEPs encoding the peroxidase were enriched in the phenylpropanoid biosynthesis pathway, which catalyzes the conversion of coumaryl alcohol to hydroxy-phenyl lignin. The decrease of the peroxidase activity inhibited lignin synthesis, which may cause plant softening under submergence stress. We also selected other pathways related to submergence stress, such as MAPK signaling pathway-plant, carbon metabolism, isoflavonoid biosynthesis and tryptophan metabolism. Our study provides a theoretical basis for a better understanding of the molecular mechanisms of submergence tolerance.

## Results

### Selection of soybean varieties by submergence tolerance

**Figure 1:**
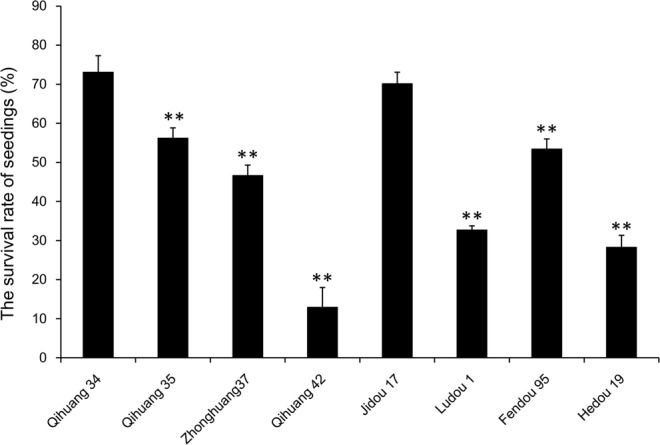
Statistical analysis of the survival rate of the seedlings for different varieties. Error bars on the boxes indicate the standard deviations of three biological replicates. **P < 0.01; Student’s *t*-test.

We selected 8 cultivated soybean varieties, including Qihuang 34, Qihuang 35, Zhonghuang 37, Qihuang 42, Jidou 17, Ludou 1, Fendou 95 and Hedou 19, and used Nannong 1138-2 (a sensitive variety) as the control. These materials were used for the submergence treatment, and the survival rate of seedlings was 73.08%, 56.19%, 46.65%, 12.92%, 70.12%, 32.71%, 53.43% and 28.26%, respectively (Fig. 1). Consequently, Qihuang 34, which showed the highest resistance to submergence stress, was used for transcriptome and proteome sequencing.

### RNA-Seq analysis

RNA sequencing (RNA-Seq) based-transcriptome was performed in order to obtain the key genes involved in submergence tolerance. The samples from soybean root tissues were used for the cDNA library construction and 23.33–23.94 million raw reads were generated by sequencing. After removing adapter sequences and low quality reads, a total of 23.18–23.78 million clean reads were generated for each sample. The ratio of clean reads to total raw reads ranged from 97.17% to 99.9%. The total mapping ratio was from 86.65% to 92.07% (Table S1). The correlation of gene expression patterns between the biologically repeated samples was consistent (Fig. S1).

### Comparative analysis of DEGs in response to the submergence treatment

**Figure 2:**
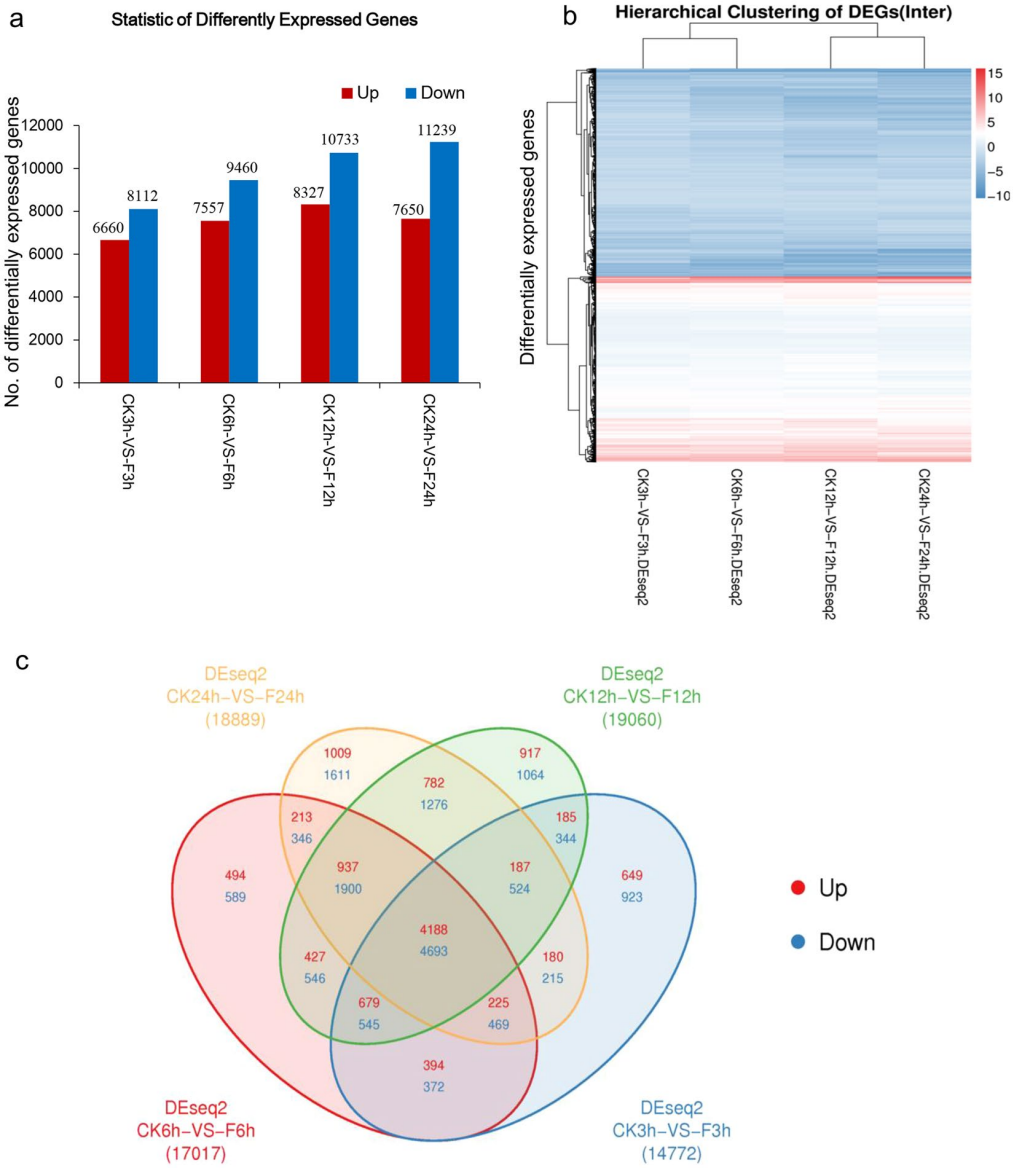
Comparison of the differentially expressed genes (DEGs) in response to submergence stress in soybean. (**a**) The number of up- and down-regulated genes under submergence conditions. (**b**) A cluster heat map of DEGs resulting from submergence treatment. Red, up-regulated genes; blue, down-regulated genes. Darker color indicates greater significance. (**c**) Venn diagram of common DEGs under 3, 6, 12 and 24-h submergence treatments in soybean seedlings. CK: Control; F: Flooding (submergence treatment).

The numbers of the up-regulated DEGs were normally distributed. The number of the genes was the least under the 3-h submergence stress, which began to increase at 6 h, reached the maximum at 12 h, and finally decreased at 24 h. However, the number of the down-regulated DEGs increased gradually with the time of submergence, and reached the maximum at 24 h (Fig. 2a). A cluster heat map indicated that the expression patterns of the DEGs were similar for the plants at these four time points (Fig. 2b). The Venn diagram clearly showed the number of the up-/down-regulated common genes at various time points under the submergence condition. A total of 4188 up-regulated and 4693 down-regulated genes were shared by the plants at these four time points (Fig. 2c). The log_2_FoldChange and other detailed information of the DEGs in all sample combinations are listed in Table S2.

### GO enrichment analysis based on transcriptome

To dissect the function of the DEGs under the submergence stress, we performed gene ontology (GO) enrichment by transcriptome analysis. The GO terms with *P* value < 0.05 were considered as enriched GO terms. GO terms for the up-/down-regulated DEGs were shown at various time points (Tables 1, 2). The up-regulated DEGs were mainly enriched in binding, phosphotransferase activity, alcohol group as acceptor, kinase activity and protein kinase activity. These GO terms enriched at four time points belonged to molecular function (MF). Transferase activity, transferring phosphorus-containing groups, adenyl nucleotide binding, adenyl ribonucleotide binding, cellular protein modification process and protein modification process were enriched under the 6, 12 and 24-h submergence stresses; these GO terms belong to MF and biological process (BP). The GO terms responding to the submergence stress in the early stage (3 h) included nucleic acid binding transcription factor activity, negative regulation of macromolecule metabolic process, nucleic acid-templated transcription, RNA biosynthetic process, carbohydrate metabolic process and regulation of protein metabolic process. The GO terms associated with signal transducer and transferase activities responded to the late submergence stress (24 h) (Table 1).

**Table 1:** GO terms (Corrected *P* Value < 0.05) of up-regulated DEGs at various time points.

GO accession	Description	Term type	Corrected *P* Value			
			3 h	6 h	12 h	24 h
GO:0001071	nucleic acid binding transcription factor activity	MF	6.10E-04			
GO:0016701	oxidoreductase activity, acting on single donors with incorporation of molecular oxygen	MF	1.65E-02			
GO:0043169	cation binding	MF	4.00E-03		3.65E-03	
GO:0043167	ion binding	MF	5.39E-03		2.05E-03	
GO:0046872	metal ion binding	MF	6.70E-03		3.42E-03	
GO:0004713	protein tyrosine kinase activity	MF			1.08E-02	
GO:0004871	signal transducer activity	MF				7.27E-03
GO:0016740	transferase activity	MF			4.05E-03	1.00E-04
GO:0005488	binding	MF	2.59E-06	3.59E-11	2.64E-10	2.14E-15
GO:0016773	phosphotransferase activity, alcohol group as acceptor	MF	2.70E-04	9.87E-10	1.08E-12	2.8E-14
GO:0016301	kinase activity	MF	9.50E-04	4.91E-09	6.49E-11	3.7E-13
GO:0004672	protein kinase activity	MF	1.66E-03	9.06E-09	1.24E-11	1.48E-12
GO:0016772	transferase activity, transferring phosphorus-containing groups	MF		9.13E-06	6.92E-06	1.91E-08
GO:0030554	adenyl nucleotide binding	MF		1.50E-04	4.20E-04	2.86E-11
GO:0032559	adenyl ribonucleotide binding	MF		2.60E-04	6.80E-04	6.03E-11
GO:0097159	organic cyclic compound binding	MF		6.80E-04		4.23E-09
GO:1901363	heterocyclic compound binding	MF		7.70E-04		5.12E-09
GO:0036094	small molecule binding	MF		1.50E-03		8.94E-11
GO:0000166	nucleotide binding	MF		3.45E-03		2.13E-10
GO:1901265	nucleoside phosphate binding	MF		3.45E-03		2.13E-10
GO:0017076	purine nucleotide binding	MF		9.00E-03		3.03E-10
GO:0032553	ribonucleotide binding	MF		1.35E-02		5.98E-10
GO:0032555	purine ribonucleotide binding	MF		1.35E-02		5.98E-10
GO:0097367	carbohydrate derivative binding	MF		1.61E-02		8.10E-10
GO:0010605	negative regulation of macromolecule metabolic process	BP	2.26E-03			
GO:0006351	transcription, DNA-templated	BP	9.42E-03			
GO:0032774	RNA biosynthetic process	BP	9.42E-03			
GO:0097659	nucleic acid-templated transcription	BP	9.42E-03			
GO:0005975	carbohydrate metabolic process	BP	9.98E-03			
GO:0010466	negative regulation of peptidase activity	BP	1.39E-02			
GO:0030162	regulation of proteolysis	BP	1.39E-02			
GO:0032269	negative regulation of cellular protein metabolic process	BP	1.39E-02			
GO:0045861	negative regulation of proteolysis	BP	1.39E-02			
GO:0051248	negative regulation of protein metabolic process	BP	1.39E-02			
GO:0052547	regulation of peptidase activity	BP	1.39E-02			
GO:0050896	response to stimulus	BP		3.91E-03		
GO:0006464	cellular protein modification process	BP		3.95E-06	1.14E-09	1.82E-12
GO:0036211	protein modification process	BP		3.95E-06	1.14E-09	1.82E-12
GO:0043412	macromolecule modification	BP		7.04E-05	1.32E-08	
GO:0044267	cellular protein metabolic process	BP			1.47E-05	
GO:0044260	cellular macromolecule metabolic process	BP			3.28E-05	
GO:0044237	cellular metabolic process	BP			1.83E-03	
GO:0019538	protein metabolic process	BP			2.96E-03	
GO:0044238	primary metabolic process	BP			5.30E-03	

GO accession, the unique serial number in Gene Ontology database.

Description, the description of GO function.

Term type, the category of this GO term. BP, biological process; MF, molecular function.

Corrected *P* Value, the corrected significant level of statistics, Corrected *P* value < 0.05 means this GO term was enriched.

Up-regulated DEGs: differentially expressed genes (the cutoff value for the log_2_FoldChange ≥ 2).

**Table 2:** GO terms (Corrected *P* Value < 0.05) of down-regulated DEGs at various time points.

GO accession	Description	Term type	Corrected *P* Value			
			3 h	6 h	12 h	24 h
GO:0015630	microtubule cytoskeleton	CC		2.00E-04	1.00E-04	3.28E-05
GO:0043228	non-membrane-bounded organelle	CC		2.31E-03	2.30E-05	5.68E-05
GO:0043232	intracellular non-membrane-bounded organelle	CC		2.31E-03	2.30E-05	5.68E-05
GO:0005856	cytoskeleton	CC		9.64E-03	1.69E-03	5.70E-04
GO:0031224	intrinsic component of membrane	CC	2.70E-12	5.30E-04	3.16E-06	4.31E-10
GO:0016020	membrane	CC	5.91E-09		2.18E-03	1.56E-06
GO:0044425	membrane part	CC	5.95E-08		1.79E-03	9.22E-07
GO:0005576	extracellular region	CC	6.47E-03			5.30E-04
GO:0009507	chloroplast	CC	2.00E-05			
GO:0044434	chloroplast part	CC	2.40E-04			
GO:0031976	plastid thylakoid	CC	4.60E-04			
GO:0031984	organelle subcompartment	CC	4.60E-04			
GO:0009534	chloroplast thylakoid	CC	1.05E-03			
GO:0004553	hydrolase activity, hydrolyzing O-glycosyl compounds	MF				9.15E-03
GO:0016787	hydrolase activity	MF				3.18E-02
GO:0016798	hydrolase activity, acting on glycosyl bonds	MF				8.08E-06
GO:0016682	oxidoreductase activity, acting on diphenols and related substances as donors, oxygen as acceptor	MF			1.19E-06	8.36E-11
GO:0016679	oxidoreductase activity, acting on diphenols and related substances as donors	MF			5.81E-06	8.60E-11
GO:0003774	motor activity	MF			2.95E-03	3.53E-02
GO:0003824	catalytic activity	MF	2.44E-10	6.89E-10	4.13E-06	5.12E-16
GO:0016491	oxidoreductase activity	MF	4.72E-03	2.00E-04		2.44E-07
GO:0005506	iron ion binding	MF	1.28E-05	3.52E-03		
GO:0016872	intramolecular lyase activity	MF		1.20E-02		
GO:0071705	nitrogen compound transport	BP	6.10E-04			
GO:0009699	phenylpropanoid biosynthetic process	BP	1.34E-03			
GO:0044550	secondary metabolite biosynthetic process	BP	1.34E-03			
GO:0006811	ion transport	BP	4.66E-03			
GO:0015833	peptide transport	BP	5.05E-03			
GO:0009808	lignin metabolic process	BP	1.24E-03		5.53E-06	5.76E-08
GO:0009698	phenylpropanoid metabolic process	BP	1.82E-08	4.80E-04	5.63E-06	4.28E-08
GO:0019748	secondary metabolic process	BP	1.82E-08	4.80E-04	5.63E-06	4.28E-08
GO:0007017	microtubule-based process	BP		1.18E-03	5.81E-05	2.46E-03
GO:0006461	protein complex assembly	BP		8.60E-04	1.56E-03	
GO:0070271	protein complex biogenesis	BP		8.60E-04	1.56E-03	
GO:0044255	cellular lipid metabolic process	BP		1.43E-02	2.59E-03	
GO:0043623	cellular protein complex assembly	BP		2.14E-03		
GO:0071822	protein complex subunit organization	BP		9.14E-03		
GO:0006829	zinc II ion transport	BP		9.64E-03		
GO:0022607	cellular component assembly	BP		9.97E-03		
GO:0034622	cellular macromolecular complex assembly	BP		1.50E-02		
GO:0016043	cellular component organization	BP			4.00E-04	
GO:0071840	cellular component organization or biogenesis	BP			6.00E-04	

GO accession, the unique serial number in Gene Ontology database.

Description, the description of GO function.

Term type, the category of this GO term. BP, biological process; CC, cellular component; MF, molecular function.

Corrected *P* Value, the corrected significant level of statistics, Corrected *P* value < 0.05 means this GO term was enriched.

Down-regulated DEG: differentially expressed genes (the cutoff value for the log_2_FoldChange ≤ −2).

The down-regulated DEGs were mainly enriched in the intrinsic component of membrane, catalytic activity, phenylpropanoid metabolic process and secondary metabolic process at four time points, which belong to cellular component (CC), MF and BP. Other GO terms related to microtubule cytoskeleton, oxidoreductase activity and lignin metabolic process were enriched at three time points. The GO terms responded to the submergence stress in the early stage (3 h), mainly included chloroplast, plastid thylakoid, organelle subcompartment and nitrogen compound transport. The GO terms related to hydrolase activity, oxidoreductase activity and motor activity responded to the late submergence stress (24 h) (Table 2).

### KEGG pathway enrichment analysis based on transcriptome

To determine the major metabolic and signal transduction pathways affected by the submergence treatment, we performed KEGG (Kyoto Encyclopedia of Genes and Genomes) enrichment analysis (Tables 3, 4).

**Table 3:** KEGG pathways (Corrected *P* Value < 0.05) of up-regulated DEGs at various time points.

KEGG pathway	Corrected *P* Value			
	3 h	6 h	12 h	24 h
Glyoxylate and dicarboxylate metabolism			1.48E-02	
MAPK signaling pathway - plant	1.06E-08	5.32E-09	1.48E-10	3.36E-05
Taurine and hypotaurine metabolism	1.66E-07	3.35E-05	1.98E-03	9.10E-04
Plant-pathogen interaction	6.77E-05	1.88E-09	5.93E-09	1.55E-11
Protein processing in endoplasmic reticulum	6.28E-04	1.06E-02	4.59E-04	4.69E-05
Plant hormone signal transduction	1.33E-03	3.57E-06	4.59E-04	1.19E-02
Glycolysis/Gluconeogenesis	5.36E-06	2.22E-02	3.06E-02	
Fatty acid degradation	3.75E-05	9.00E-03	3.06E-02	
Circadian rhythm - plant		2.48E-02	4.59E-04	2.04E-03
Ubiquitin mediated proteolysis			3.38E-03	3.65E-04
RNA transport				2.45E-03
Inositol phosphate metabolism	2.45E-03			3.99E-03
Benzoxazinoid biosynthesis	3.63E-02			
Biosynthesis of secondary metabolites	3.03E-03			
Metabolic pathways	3.67E-03			
Glyoxylate and dicarboxylate metabolism	2.77E-02			
Phosphatidylinositol signaling system	3.74E-03	3.85E-02		
Carbon metabolism	5.24E-05	3.85E-02		
Valine, leucine and isoleucine degradation	8.54E-04	3.85E-02		
Photosynthesis		3.85E-02		

Corrected *P* Value, the corrected significant level of statistics, Corrected *P* value < 0.05 means this GO term was enriched.

Up-regulated DEGs: differentially expressed genes (the cutoff value for the log_2_FoldChange ≥ 2).

**Table 4:** KEGG pathways (Corrected *P* Value < 0.05) of down-regulated DEGs at various time points.

KEGG pathway	Corrected *P* Value			
	3 h	6 h	12 h	24 h
Pyruvate metabolism				8.22E-06
Glycolysis/Gluconeogenesis				4.36E-03
Amino sugar and nucleotide sugar metabolism				6.96E-03
AGE-RAGE signaling pathway in diabetic complications			1.81E-02	6.45E-03
Arginine and proline metabolism		4.35E-03	1.85E-02	2.39E-03
Ascorbate and aldarate metabolism		9.17E-03	2.34E-02	1.32E-07
Biosynthesis of secondary metabolites	8.24E-10	2.51E-09	2.52E-02	1.10E-14
Carotenoid biosynthesis	1.32E-06	1.31E-04	5.59E-03	1.55E-03
Metabolic pathways	6.69E-04	5.99E-03	1.07E-03	2.14E-10
beta-Alanine metabolism	1.99E-02	9.17E-03	2.63E-02	1.19E-04
Isoflavonoid biosynthesis	3.00E-12	3.10E-09		1.81E-08
Phenylpropanoid biosynthesis	1.95E-08	9.17E-03		5.37E-08
Flavonoid biosynthesis	6.52E-06	3.20E-03		1.55E-03
Flavone and flavonol biosynthesis	4.29E-04	9.33E-04		1.46E-03
MAPK signaling pathway - plant	1.87E-03	1.27E-02		
Anthocyanin biosynthesis	3.33E-03	2.12E-02		
Sesquiterpenoid and triterpenoid biosynthesis	9.21E-03	2.03E-02		
Plant hormone signal transduction	2.48E-05			
Stilbenoid, diarylheptanoid and gingerol biosynthesis	2.90E-04			
Phenylalanine metabolism	1.22E-02			
Galactose metabolism	1.95E-02			5.41E-04
Fatty acid biosynthesis		3.40E-03	5.59E-03	
Riboflavin metabolism			5.59E-03	
Propanoate metabolism			2.79E-02	
Base excision repair			3.03E-02	
Fatty acid metabolism			4.49E-03	
Vancomycin resistance			9.35E-03	

Corrected *P* Value, the corrected significant level of statistics, Corrected *P* value < 0.05 means this GO term was enriched.

Down-regulated DEG: differentially expressed genes (the cutoff value for the log_2_FoldChange ≤ −2).

Five main KEGG pathways were enriched at four time points for the up-regulated DEGs, including MAPK signaling pathway-plant, taurine and hypotaurine metabolism, plant-pathogen interaction, protein processing in endoplasmic reticulum and plant hormone signal transduction. Glycolysis/gluconeogenesis and fatty acid degradation were enriched under the 3, 6, and 12-h submergence stresses. Circadian rhythm-plant was enriched under the 6, 12 and 24-h submergence stresses. Inositol phosphate metabolism, benzoxazinoid biosynthesis, biosynthesis of secondary metabolites, metabolic pathways, glyoxylate and dicarboxylate metabolism responded to the submergence stress in the early stage (3 h), while ubiquitin mediated proteolysis and RNA transport responded to the submergence stress in the late stage (24 h) (Table 3).

A total of twenty-seven KEGG pathways were enriched due to the down-regulated DEGs. Biosynthesis of secondary metabolites, carotenoid biosynthesis, metabolic pathways and beta-Alanine metabolism were enriched at these four time points. Six KEGG pathways were enriched at three time points, including arginine and proline metabolism, ascorbate and aldarate metabolism, isoflavonoid biosynthesis, phenylpropanoid biosynthesis, flavonoid biosynthesis and flavone and flavonol biosynthesis. Plant hormone signal transduction, stilbenoid, diarylheptanoid and gingerol biosynthesis, and phenylalanine metabolism responded to the submergence stress in the early stage (3 h), while pyruvate metabolism, glycolysis/gluconeogenesis, amino sugar and nucleotide sugar metabolism responded to the submergence stress in the late stage (24 h) (Table 4).

### GO enrichment analysis based on transcriptome and proteome

Only the samples under the 24-h submergence treatment were taken for the proteome sequencing analysis (Fig. S2, Table S3). The result demonstrated that the GO terms in the up-regulated DEGs/DEPs were mainly enriched in cation binding, metal ion binding, ion binding and carbohydrate metabolic process, which belong to MF and BP (Table 5).

**Table 5:** GO terms (Corrected *P* Value < 0.05) for the up-regulated DEGs/DEPs by association analysis of transcriptome and proteome.

GO accession	Description	Term type	Corrected *P* Value	
			Transcriptome	Proteome
GO:0043169	cation binding	MF	2.26E-03	4.84E-07
GO:0046872	metal ion binding	MF	2.79E-03	7.56E-08
GO:0043167	ion binding	MF	6.89E-03	2.74E-02
GO:0005975	carbohydrate metabolic process	BP	1.36E-02	8.74E-03

GO accession, the unique serial number in Gene Ontology database.

Description, the description of GO function.

Term type, the category of this GO term. BP, biological process; MF, molecular function.

Corrected *P* Value, the corrected significant level of statistics, Corrected *P* value < 0.05 means this GO term was enriched.

Up-regulated DEGs: differentially expressed genes (the cutoff value for the log_2_FoldChange ≥ 2).

Up-regulated DEPs: up-regulated differentially expressed proteins (the cutoff value for the log_2_FoldChange > 0.26).

The GO terms in the down-regulated DEGs/DEPs were mainly enriched in membrane, membrane part, oxidoreductase activity, oxidoreductase activity, acting on diphenols and related substances as donors, oxygen as acceptor, oxidoreductase activity, acting on diphenols and related substances as donors, phenylpropanoid metabolic process and secondary metabolic process (Table 6).

**Table 6:** GO terms (Corrected *P* Value < 0.05) for the down-regulated DEGs/DEPs by association analysis of transcriptome and proteome.

GO accession	Description	Term type	Corrected *P* Value	
			Transcriptome	Proteome
GO:0016020	membrane	MF	2.80E-04	2.53E-02
GO:0044425	membrane part	MF	6.00E-04	1.49E-02
GO:0016491	oxidoreductase activity	MF	8.41E-03	5.85E-04
GO:0016682	oxidoreductase activity, acting on diphenols and related substances as donors, oxygen as acceptor	MF	1.12E-02	3.41E-02
GO:0016679	oxidoreductase activity, acting on diphenols and related substances as donors	MF	2.02E-02	1.91E-02
GO:0009698	phenylpropanoid metabolic process	BP	2.58E-05	5.03E-03
2GO:0019748	secondary metabolic process	BP	2.58E-05	2.05E-02

GO accession, the unique serial number in Gene Ontology database.

Description, the description of GO function.

Term type, the category of this GO term. BP, biological process; MF, molecular function.

Corrected *P* Value, the corrected significant level of statistics, Corrected *P* value < 0.05 means this GO term was enriched.

Down-regulated DEG: differentially expressed genes (the cutoff value for the log_2_FoldChange ≤ −2).

Down-regulated DEPs: down**-**regulated differentially expressed proteins (the cutoff value for the log_2_FoldChange< −0.26).

### KEGG pathway enrichment analysis based on transcriptome and proteome

The analysis of the KEGG pathways is the same as that of the GO terms (Fig. S3). The result showed that five KEGG pathways in the up-regulated DEGs were enriched, and twenty-two DEGs/DEPs were identified under the submergence stress. All DEGs/DEPs involved in glycolysis/gluconeogenesis, carbon metabolism, MAPK signaling pathway-plant, fatty acid degradation and plant-pathogen interaction were up-regulated simultaneously. The number of DEGs/DEPs enriched in glycolysis/gluconeogenesis pathway was the highest (Table 7).

**Table 7:** KEGG pathways (Corrected *P* Value < 0.05) for the up-regulated DEGs/DEPs by assocation analysis of transcriptome and proteome.

Pathways	DEGs/DEPs	Corrected *P*-value†	
		Transcriptome	Proteome
MAPK signaling pathway - plant	Glyma.05G124000, Glyma.10G152200 Glyma.05G123700, Glyma.05G123900	1.49E-04	5.57E-10
Plant-pathogen interaction	Glyma.05G124000, Glyma.08G078900 Glyma.05G123900	1.87E-02	4.33E-06
Glycolysis/Gluconeogenesis	Glyma.07G153100, Glyma.02G222400 Glyma.19G000700, Glyma.09G153900 Glyma.04G240800, Glyma.18G219100 Glyma.08G165400, Glyma.19G017200 Glyma.03G055100, Glyma.04G213900 Glyma.18G204200	4.36E-10	1.30E-02
Fatty acid degradation	Glyma.03G221400	1.28E-02	2.71E-02
Carbon metabolism	Glyma.10G201100, Glyma.15G262100 Glyma.03G244800, Glyma.16G041200 Glyma.16G204600	3.67E-08	3.83E-02

Corrected *P* Value, the corrected significant level of statistics, Corrected *P* value < 0.05 means this GO term was enriched.

DEGs/DEPs, differentially expressed genes/proteins.

Up-regulated DEGs: differentially expressed genes (the cutoff value for the log_2_FoldChange ≥ 2).

Up-regulated DEPs: up-regulated differentially expressed proteins (the cutoff value for the log_2_FoldChange > 0.26).

Six KEGG pathways in the down-regulated DEGs/DEPs were enriched, and thirty-eight DEGs/DEPs were identified under the submergence stress. The down-regulated DEGs/DEPs were related to phenylpropanoid biosynthesis, tryptophan metabolism, metabolic pathways, isoflavonoid biosynthesis, sesquiterpenoid and triterpenoid biosynthesis and biosynthesis of secondary metabolites (Table 8).

**Table 8:** KEGG pathways (Corrected *P* Value < 0.05) for the down-regulated DEGs/DEPs by assocation analysis of transcriptome and proteome.

Pathways	DEGs/DEPs	Corrected *P*-value†	
		Transcriptome	Proteome
Phenylpropanoid biosynthesis	Glyma.06G275900, Glyma.20G214200, Glyma.02G259300, Glyma.04G227200 Glyma.01G163100, Glyma.12G195500 Glyma.20G180800, Glyma.14G053600 Glyma.03G039800	3.39E-05	3.69E-13
Tryptophan metabolism	Glyma.06G176200, Glyma.08G350800	2.61E-02	8.30E-07
Metabolic pathways	Glyma.08G074700, Glyma.07G040100 Glyma.08G093300, Glyma.06G275900 Glyma.20G214200, Glyma.02G259300 Glyma.06G176200, Glyma.04G227200 Glyma.09G070000, Glyma.05G216400 Glyma.03G021200, Glyma.13G336600 Glyma.19G028400, Glyma.08G350800 Glyma.12G020000, Glyma.12G038200 Glyma.07G003400, Glyma.01G163100 Glyma.11G171400, Glyma.12G195500 Glyma.17G164100, Glyma.08G200200 Glyma.09G239800, Glyma.20G180800 Glyma.07G233800, Glyma.14G053600 Glyma.03G039800, Glyma.18G265300	3.69E-05	3.59E-04
Isoflavonoid biosynthesis	Glyma.08G246700,Glyma.18G258000	8.05E-06	5.29E-04
Sesquiterpenoid and triterpenoid biosynthesis	Glyma.08G350800, Glyma.12G038200	2.61E-02	1.97E-03
Biosynthesis of secondary metabolites	Glyma.07G040100, Glyma.06G275900 Glyma.20G214200, Glyma.02G259300 Glyma.06G176200, Glyma.04G227200 Glyma.09G070000, Glyma.03G021200 Glyma.19G028400, Glyma.08G350800 Glyma.12G038200, Glyma.01G163100 Glyma.11G171400, Glyma.12G195500 Glyma.17G164100, Glyma.18G285800 Glyma.09G239800, Glyma.20G180800 Glyma.07G233800, Glyma.14G053600 Glyma.03G039800, Glyma.18G265300	2.26E-12	2.29E-03

Corrected *P* Value, the corrected significant level of statistics, Corrected *P* value < 0.05 means this GO term was enriched.

DEGs/DEPs, differentially expressed genes/proteins.

Down-regulated DEG: differentially expressed genes (the cutoff value for the log_2_FoldChange ≤ −2).

Down-regulated DEPs: down-regulated differentially expressed proteins (the cutoff value for the log_2_FoldChange < −0.26).

### Submergence stress activated the expression of glycolysis/gluconeogenesis related genes/proteins

**Figure 3:**
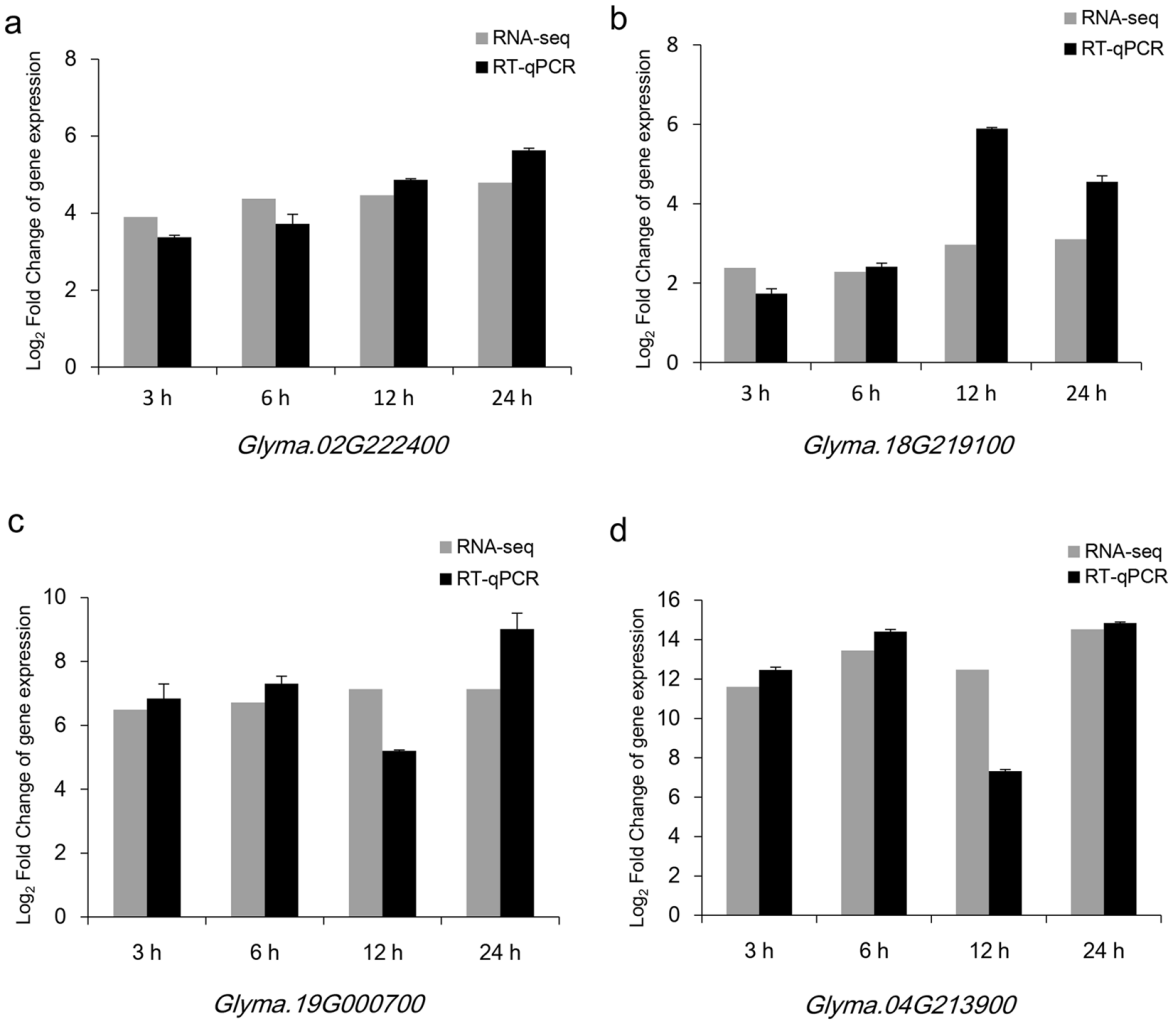
Expression patterns of four representative genes involved in glycolysis/gluconeogenesis were determined by RNA-Seq and RT-qPCR at 3, 6, 12 and 24-h submergence stresses. Normalization for RT-qPCR was performed using the *ELF1B* gene as an internal control, and the gene expression levels in the submergence treatment samples are indicated by the log_2_FoldChange relative to that of the corresponding control samples. Error bars on the black boxes indicate the standard deviations of three biological replicates of RT-qPCR analyses. (**a**–**d**) Genes related to glycolysis/gluconeogenesis.

A total of eleven DEGs/DEPs from the glycolysis/gluconeogenesis pathway were isolated by the transcriptome and proteome analyses. All genes encoding the key enzymes in the glycolysis/gluconeogenesis pathway were up-regulated. RT-qPCR was used to verify the RNA-Seq data. Four genes showing different levels of expression were selected, including *Glyma*.*02G222400*, *Glyma*.*18G219100*, *Glyma*.*19G000700* and *Glyma*.*04G213900* (Table 7), which encode fructose-bisphosphate aldolase (FBA), pyruvate kinase (PK), phosphoglycerate mutase (PGAM) and alcohol dehydrogenase (ADH), respectively. The expression levels were analyzed by RT-qPCR using the gene-specific primers. The results demonstrated that the expressions of these genes were very similar to those determined by RNA-Seq, which were up-regulated at four time points. The log_2_FoldChange of gene expression of *Glyma*.*04G213900* in the submergence-treatment group was more than 11-fold higher than that of the control and maintained at a high level at four time points, except the time point at 12 h (Fig. 3).

### Submergence stress repressed the expression of phenylpropanoid biosynthesis-related genes/proteins

**Figure 4:**
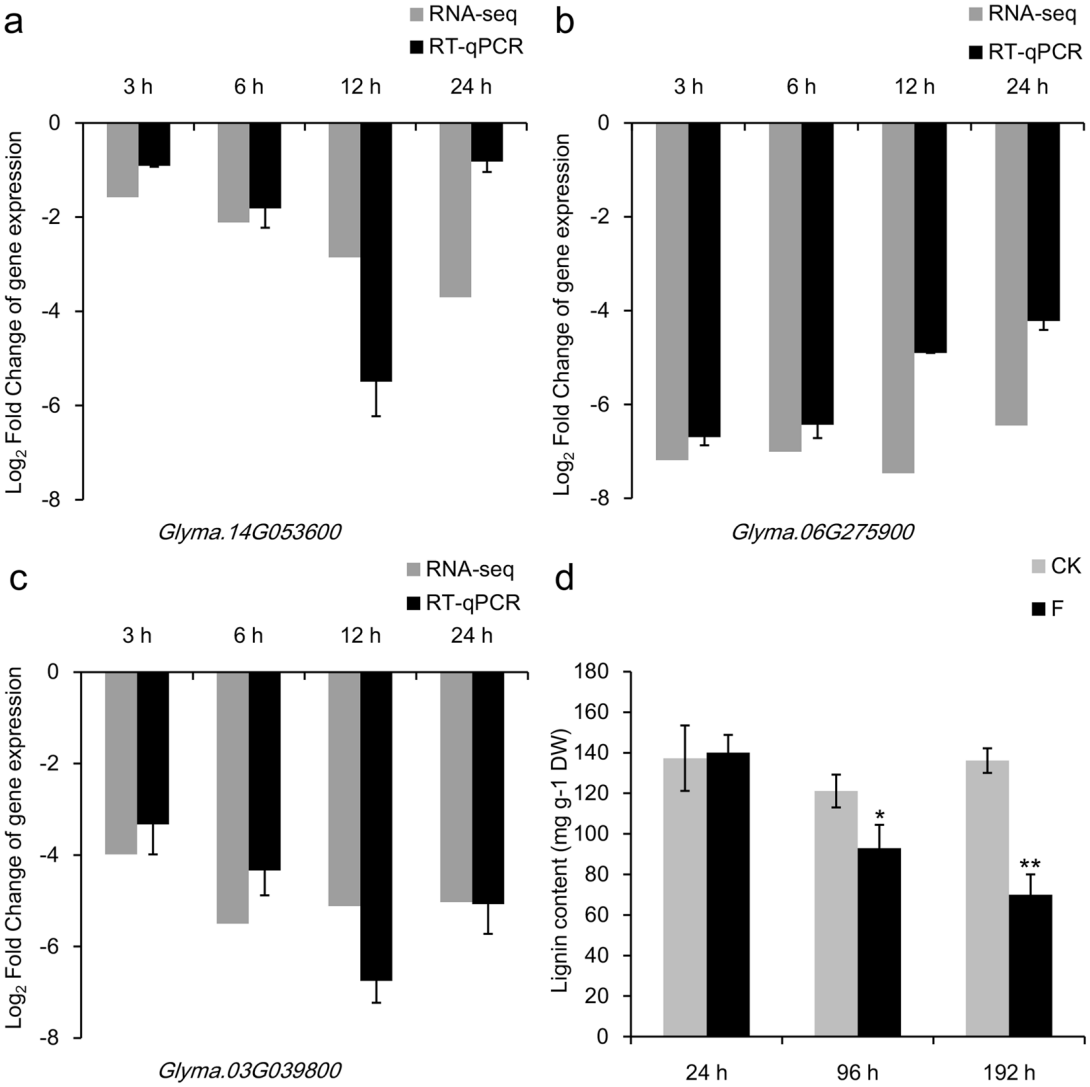
The expression patterns of genes involved in biosynthesis of phenylpropanoid and lignin content of soybean roots. (**a**–**c**) Expression patterns of three representative genes involved in phenylpropanoid biosynthesis were determined by RNA-Seq and RT-qPCR at the 3, 6, 12 and 24-h submergence stresses. Normalization for RT-qPCR was performed using the *ELF1B* gene as an internal control. The gene expression levels in the submergence treatment samples are indicated by the log_2_FoldChange relative to that of the corresponding control samples. Error bars on the black boxes indicate the standard deviations of three biological replicates of RT-qPCR analyses. (**a**–**c**) genes related to phenylpropanoid biosynthesis. (**d**) The lignin content of soybean roots under the submergence treatment and without the submergence treatment. The sampling time points are 24, 96 and 192 h under the submergence stress. Error bars on the boxes indicate the standard deviations of three biological replicates. *P < 0.05, **P < 0.01; Student’s *t*-test. CK: Control; F: Flooding (submergence treatment).

Nine DEGs/DEPs from the phenylpropanoid biosynthesis pathway were isolated by the transcriptome and proteome analyses, all of which were down-regulated simultaneously (Table 8). Out of these nine DEGs, eight genes encode peroxidase, while *Glyma*.*20G180800* encodes phenylalanine ammonia-lyase 2. We selected three genes to validate the results of RNA-Seq, among which, *Glyma*.*14G053600* and *Glyma*.*06G275900* encode peroxidase P7, and *Glyma*.*03G039800* encodes cationic peroxidase 1. These genes were down-regulated at four time points as shown by RT-qPCR analysis, which is consistent with that of RNA-Seq (Fig. 4a–c). We further measured the lignin content, which showed no difference between the 24-h submergence treatment group and the control group. The lignin content was remarkably different at 96 h, and the difference was significantly increased at 192 h between the submergence treatment group and the control group. The content of lignin decreased with the duration of the submergence treatment (Fig. 4d).

### Submergence stress induced the expression of other pathways-related genes/proteins

**Figure 5:**
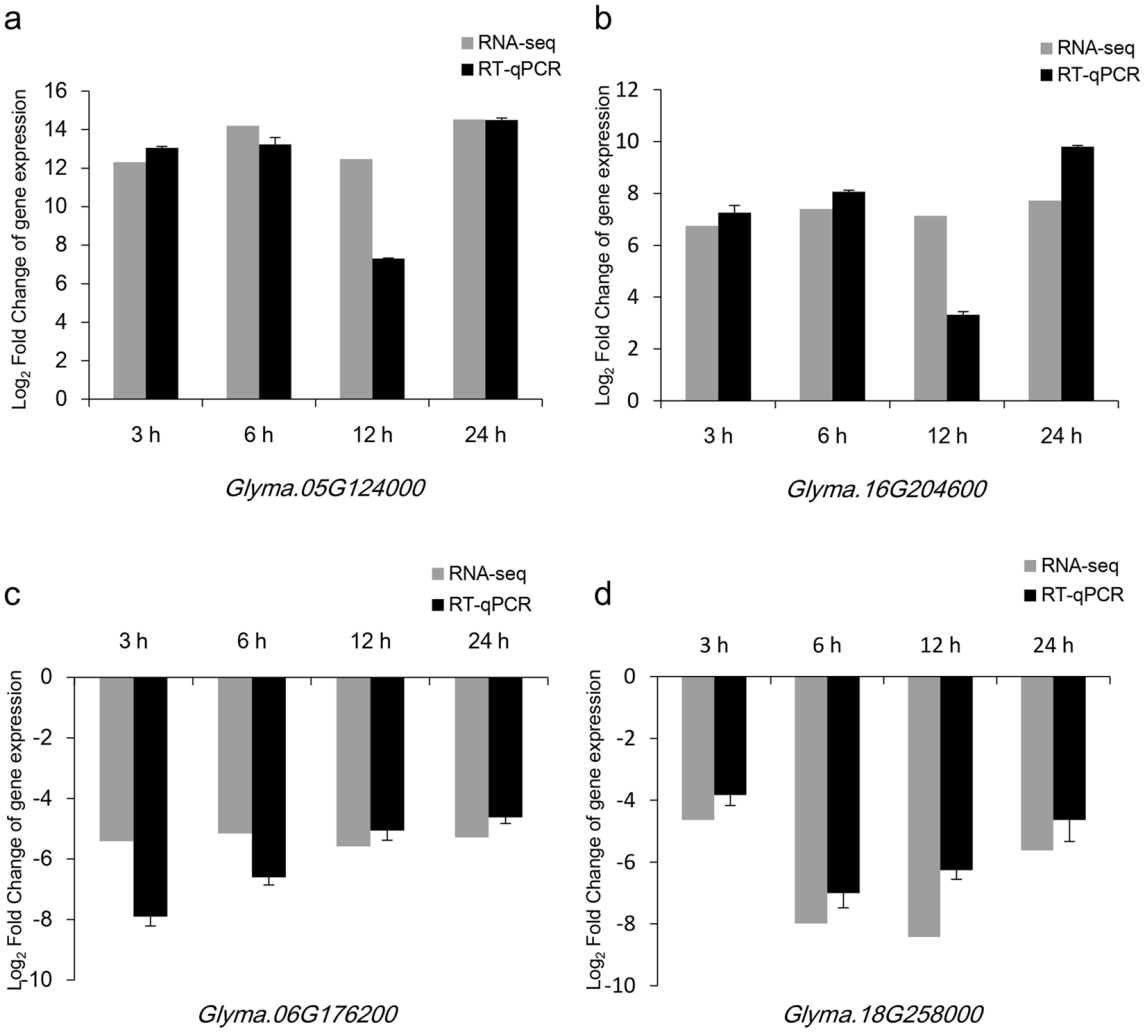
Expression patterns of four representative genes involved in other pathways were determined by RNA-Seq and RT-qPCR at the 3, 6, 12 and 24-h submergence stresses. Normalization for RT-qPCR was performed using the *ELF1B* gene as an internal control, and the gene expression levels in the submergence treatment samples are indicated by the log_2_FoldChange relative to that of the corresponding control samples. Error bars on the black boxes indicate the standard deviations of three biological replicates of RT-qPCR analyses. (**a**) Gene related to MAPK signaling pathway-plant; (**b**) gene related to carbon metabolism; (**c**) gene related to tryptophan metabolism; (**d**) gene related to isoflavonoid biosynthesis.

We also selected representative genes from the other pathways according to the expression levels in RNA-Seq. The expression analysis was performed for the selected genes, *Glyma*.*05G124000*, *Glyma*.*16G204600 Glyma*.*06G176200* and *Glyma*.*18G258000*, which belong to MAPK signaling pathway-plant, carbon metabolism, tryptophan metabolism and isoflavonoid biosynthesis. *Glyma*.*05G124000*, *Glyma*.*16G204600*, *Glyma*.*06G176200* and *Glyma*.*18G258000* encode polygalacturonase inhibitor, enolase, cytochrome P450 71A1 and malonyl-CoA: anthocyanidin 5-O-glucoside-6″-O-malonyltransferase, respectively. The comparative analysis of these genes showed that the expression patterns in the RT-qPCR analysis were similar to those observed in the RNA-Seq data, in which *Glyma*.*05G124000* and *Glyma*.*16G204600* were up-regulated, while *Glyma*.*06G176200* and *Glyma*.*18G258000* were down-regulated at four time points under the submergence stress (Fig. 5). This result confirmed that RNA-Seq and our experimental results were reliable.

## Discussion

### Submergence stress activated carbon metabolism

Because the oxygen-dependent respiration is greatly limited under submergence conditions, the acceleration of carbohydrate metabolism is critical for plant survival^21^. Many crops, including soybean, are sensitive to flooding stress^22^. Under flooding conditions, oxygen is insufficient for normal energy generation in plants, and glycolysis/gluconeogenesis becomes the main way for plants to obtain energy^2^. Some studies have shown that flooding of soybean seedlings can increase the abundance of proteins in glycolysis and fermentation^11,14,19,23^. Enzyme-encoding genes participating in the glycolysis/gluconeogenesis pathways have been isolated in the early stage of flooding^15,24–27^. DEGs encoding glucose-6-phosphate isomerase (GPI), 6-phosphofructokinase (6-PFK), glyceraldehyde-3-phosphate dehydrogenase (GAPDH), FBA, PGAM, and PK are up-regulated in *Sesbania cannabina*^15^. Several DEGs involved in glycolysis and alcohol fermentation are significantly accumulated in both ‘Zaoer-N’ and ‘Pepino’ in cucumber, including 6-phosphogluconate dehydrogenase (G6PD), triose phosphate isomerase (TPI), pyruvate decarboxylase (PDC), PFK, ADH, and PK^27^. Proteins involved in glycolysis/fermentation are increased under flooding stress, such as enolase and ADH^28^. ADH is an important enzyme involved in alcohol fermentation, and has been observed in many plant species such as maize^29^, sorghum^30^, and *Sesbania cannabina*^15^. The maize mutant with a deficient *ADH* gene is more sensitive to flooding than the wild type plants^29^. The ADH activity in the flood-tolerant sorghum cultivar SSG-59-3 is significantly higher than that of the sensitive variety S-308^30^. Five up-regulated DEGs encoding ADH involved in alcohol fermentation have been identified, while none of these DEGs show a changed expression in the late stage of flooding in *Sesbania cannabina*^15^. The expression of *ADH* is specifically enhanced in waterlogged cotton, suggesting that *ADH* may play an important role in sustaining cotton growth under waterlogging stress^31^.

In this study, the number of DEGs/DEPs in the glycolysis/gluconeogenesis pathway was the highest. These DEGs/DEPs encode key enzymes of this pathway under submergence stress, such as FBA, PK, PGAM, GPI, ADH, PDC, enolase, and phosphoglycerate kinase (PGK). *Glyma*.*02G222400*, *Glyma*.*18G219100*, *Glyma*.*19G000700* and *Glyma*.*04G213900* encode FBA, PK, PGAM, and ADH, respectively. These enzyme-encoding genes isolated from our study were new genes, and the up-regulated expressions under the submergence stress were confirmed by RT-qPCR, suggesting that they responded to the submergence stress continuously (Fig. 3). Two proteins were previous reported, including Glyma.15g262100 and Glyma.08g165400 (Table S4). In addition, the GO terms of the up-regulated DEGs related to phosphotransferase activity and alcohol group as acceptor were enriched at four time points by transcriptome analyses (Table 1), and carbohydrate metabolic process was enriched by the transcriptome and proteome analyses (Table 5). Thus, the submergence treatment might activate the glycolysis/gluconeogenesis pathway, and the expressions of the enzyme-encoding genes might lead to the production of ATP to maintain the plant survival (Fig. S4). Previous reports show enzyme-encoding DEGs participating in the glycolysis/gluconeogenesis pathways respond to the early stage of flooding^15,24–27^. However, our result showed that the related genes involved in glycolysis/gluconeogenesis were induced to express persistently at four time points for improving the tolerance in Qihuang 34 under submergence stress. The reason was maybe the differences of the species and genetic background of the plants.

### Submergence stress repressed lignin biosynthesis

Phenylpropanoid biosynthesis is regulated by biotic and abiotic stimuli, and phenylpropanoid-based polymers such as lignin, suberin, and tannin contribute substantially to the stability and robustness of plants in the face of mechanical or environmental damages. Lignin plays an important role in mechanical support, water transportation and resistance to the harmful environment for plants^32^. In our study, the GO terms of the down-regulated DEGs related to phenylpropanoid metabolic process and lignin metabolic process were enriched at various time points (Table 2).

Previous reports have demonstrated that genes related to phenylpropanoid biosynthesis are differentially expressed, and many genes have been identified^15,31^. Our experiment identified nine DEGs/DEPs related to phenylpropanoid biosynthesis by the transcriptome and proteome analyses under submergence stress (Table 8). All of these DEGs/DEPs were down-regulated simultaneously at four time points by RNA-Seq analyses. Eight of these genes encoded peroxidase which catalyzes the conversion of coumaryl alcohol to hydroxy-phenyl lignin in the final step of lignin biosynthesis (Fig. S5). Three key genes were selected based on the differential expression levels. The expression data and pattern revealed by RT-qPCR were highly consistent with those obtained by RNA-Seq (Fig. 4a–c). We further measured the lignin content, and the result showed no difference between the submergence and control group under the 24-h submergence stress. A highly significant difference was observed at 192 h between the submergence treatment group and the control group. The content of lignin decreased with the time of the submergence treatment (Fig. 4d). The decrease of the gene expression inhibited lignin biosynthesis, which might cause plant softening under the submergence treatment for a long period of time.

### Other KEGG pathways under submergence stress

To date, few reports related to isoflavones and flooding stress have been conducted. Previous reports indicated that four of twenty-eight differentially expressed genes related to flavonoid biosynthesis are down-regulated under waterlogging in cotton^31^. Two DEGs/DEPs participating in isoflavonoid biosynthesis were down-regulated at various time points in our experiment (Table 8). A gene (*Glyma*.*18G258000*) with the highest expression change in RNA-Seq was selected and subjected to RT-qPCR; the results were consistent with that of RNA-Seq (Fig. 5). *Glyma*.*18G258000* encodes malonyl-CoA: anthocyanidin 5-O-glucoside- 6″-O-malonyltransferase-like, which catalyzes the transformation of (daidzein, glycitein, biochanin and genistein) 7-O-glucoside to (daidzein, glycitein, biochanin and genistein) 7-O-glucoside-6″-malonate (Fig. S6). The decreased expression of *Glyma*.*18G258000* caused the accumulations of (daidzein, glycitein, biochanin and genistein) 7-O-glucoside. According to previous reports, the concentrations of biochanin A-7-O-glucoside, and genistein-7-O-glucoside in the leaves increase by two/three folds in response to waterlogging^33^. Consequently, we predicted that soybean might protect themselves against the anaerobic environment by accumulating (daidzein, glycitein, biochanin and genistein) 7-O-glucoside under submergence stress.

Increasing evidence has shown that MAPKs play key roles in plant signal transduction in response to drought, salinity, cold, and wounding^34–40^. NtMEK2 is a salicylic acid-induced protein kinase (SIPK)/wounding-induced protein kinase (WIPK) in tobacco^41^. MAPK cascade (MEKK1, MPK3/MPK6 and MKK4/MKK5), together with its upstream receptor kinase FLS2 and downstream transcription factors WRKY22/WRKY29 have been characterized in *Arabidopsis*. MAPKs are important signal transduction components in plant defense responses^42^. The OsMAPK5 protein possesses kinase activity, which is activated by cold, drought, and salinity stresses^43^.

Activation of MAPKs has been rarely observed in plants exposed to flooding stress. In our study, we identified four genes (*Glyma*.*05G124000*, *Glyma*.*10G152200*, *Glyma*.*05G123700* and *Glyma*.*05G123900*) related to the MAPK signaling pathway -plant under submergence stress. *Glyma*.*10G152200* encodes a respiratory burst oxidase homolog protein B-like, and *Glyma*.*05G124000*, *Glyma*.*05G123700* and *Glyma*.*05G123900* encode polygalacturonase inhibitor 2. These three genes were also identified in the plant-pathogen interaction pathway (Table 7). Glyma.05G123900 was the same as that discovered in previous reports (Table S4). The DEGs/DEPs isolated from the MAPK signaling pathway were up-regulated under submergence stress. We analyzed the expression level of *Glyma*.*05G124000*, which showed a consistent result compared with the RNA-Seq data (Fig. 5). According to the map of the MAPK signaling pathway, we found *Glyma*.*05G124000*, *Glyma*.*05G123700* and *Glyma*.*05G123900* encoded the same protein, FLS2. Therefore, we predict that submergence stress triggers basal defense responses similar to pathogen attack, and the transmission of signals through FLS2 further activates the MAPK signaling pathway (Fig. S7).

Two genes involved in tryptophan metabolism, *Glyma*.*06G176200* and *Glyma*.*08G350800*, were isolated, which encode cytochrome P450 71A1 and cytochrome P450 93E1, respectively. These genes were down-regulated at four time points as revealed by the transcriptome data. *Glyma*.*06G176200* was selected for RT-qPCR verification, and the results were consistent with that of RNA-Seq (Fig. 5). Inhibition of cytochrome P450 77A1 may enhance soybean tolerance to flooding stress^13^. Cytochrome P450 71A1, 93E1 and 77A1 belong to the same protein family, indicating the decreased expression of cytochrome P450 71A1 and cytochrome P450 93E1 may also enhance soybean submergence tolerance.

## Conclusion

In the present study, the RNA-Seq technology was used to analyze the DEGs of soybeans subjected to 3, 6, 12 and 24-h submergence stresses, and the iTRAQ technology was used to analyze the DEPs subjected to the 24-h submergence stress. Transcriptome and proteome analyses were performed, which revealed many key DEGs/DEPs and metabolic pathways responding to submergence stress. Eleven up-regulated enzyme-encoding DEGs/DEPs involved in glycolysis/gluconeogenesis were isolated, suggesting that the glycolysis/gluconeogenesis pathway was activated for ATP production for plant survival. Eight down-regulated peroxidase encoding DEGs/DEPs related to phenylpropanoid biosynthesis were identified, which catalyzes the conversion of coumaryl alcohol to hydroxy-phenyl lignin. We measured the lignin content, which showed no difference between the 24-h submergence treatment group and the control group. However, a highly significant difference was observed at 192 h between the submergence treatment group and the control group. The content of lignin decreased with the time of submergence treatment. The decreased expression of these genes inhibited lignin biosynthesis and accumulation, which might cause plant softening under submergence stress. Other up-/down-regulated pathways and DEGs/DEPs related to submergence tolerance were identifed, such as carbon metabolism, MAPK signaling pathway-plant, fatty acid degradation and isoflavonoid biosynthesis. The present study provides a foundation for future genomic studies on submergence tolerance of soybean.

## Methods

### Plant materials and stress conditions

The focus on the flooding tolerance of Qihuang 34 was from the field. Our previous study showed that Qihuang 34 possesses stronger tolerance to flooding during the entire growth stages^44^. Then, we selected 8 cultivated soybean varieties, including Qihuang 34, Qihuang35, Zhonghuang37, Qihuang 42, Jidou 17, Ludou 1, Fendou 95, and Hedou 19, and Nannong 1138-2 (a sensitive variety) was used as the control. Seeds were sterilized in 1% sodium hypochlorite for 30 min, rinsed with distilled water several times, and then sown on the sandy soil. Ten seeds were planted in each pot (180-mm length × 140-mm width × 45-mm depth). A total of ten pots were sowed. The seedlings were grown in a psychometric room illuminated by a photoperiod of 16/8 h light/dark at 25 °C. Five seedlings with the same size were retained in each pot, and each variety eventually retained ten pots. For the submergence treatment, when two true leaves were fully unfolded, the plants were transferred to the white plastic containers filled with water. When the death rate of the seedlings of Nannong 1138-2 increased to 85%, water was released. The survival rate of the seedlings was calculated after de-submergence and seven days recovery. The seedlings without any green leaves were treated as dead seedlings. This experiment was repeated three times. The survival rate of the seedlings was the average of these three replicates.

Qihuang 34 was used as the material for transcriptome and proteome sequencing, and the submergence treatment was the same as above. The samples were collected at 3, 6, 12 and 24 h, respectively, and the untreated plants were used as the control. The root of the seedlings were collected from the control and the submergence treated group, frozen in liquid nitrogen and stored at −80 °C. Three biological replicates were performed for each sample, which contained 10 roots from 10 independent plants.

### RNA isolation

Total RNA was isolated from the root samples of the control and the treated seedlings using TRIzol reagent (Invitrogen, Carlsbad, CA USA). We determined the total RNA through Agilent 2100 Bioanalyzer (Agilent Technologies, USA). The RNA concentration was measured by a NanoDrop 1000 Spectrophotometer (Thermo Scientific, Wilmington, Delaware, USA).

### Library construction and sequencing

cDNA library construction and sequencing were performed at Beijing Genomics Institute (BGI). The mRNA with the polyA tail was isolated using oligo(dT) attached with magnetic beads, and fragmented by a fragmentation buffer. The double-strand cDNAs were synthesized by random hexamers, RNase H and DNA Polymerase I. These cDNA fragments were added with a single ‘A’ base and subsequently ligated with the adapter. The products were then purified and enriched with PCR amplification. The double-stranded PCR products were heat denatured and circularized by the splint oligo sequence. The single strand DNA circles (ssDNA circles) were collected and used for the final library. The cDNA libraries were used for sequencing with the sequencing platform BGISEQ-500 (BGI).

### Read mapping to soybean reference genome and expression analyses

Clean reads were obtained by removing reads containing adapters, unknown base with the N content greater than 10% and low quality reads. The filtered clean reads were saved as the FASTQ format. HISAT^45^ was used to map clean reads to the genome of Glycine max Wm82.a2.v1. We used Bowtie2^46^ to map the clean read to the reference sequence in order to count the rate of gene alignment, and then calculated the expression of genes and transcripts using the RSEM software package^47^.

### DEGs, GO and KEGG enrichment analyses

Prior to identifying the DEGs associated with submergence tolerance in soybean, the gene expression levels in different samples were calculated using FPKM (Fragments per kilobase for a million reads). DEGs analyses (three biological replicates per condition) were performed using the DESeq. 2 package. The DESeq. 2 method was based on the negative two term distribution principle. The DEGs were detected according to the method described by Love *et al*.^48^. The *P*-values were adjusted using the Benjamini and Hochberg approach^49^. Genes with the corrected *P* value < 0.05 were considered differentially expressed.

Gene ontology (GO) and KEGG^50^ enrichment analyses of DEGs were implemented by the GOseq R package and KOBAS2.0 software (http://kobas.cbi.pku.edu.cn), respectively. The GO terms and KEGG pathways with *P* value < 0.05 were considered as enriched GO terms and KEGG pathways, respectively.

### RT-qPCR

The gene-specific primers were designed using the PRIMER5 software (Applied Biosystems) (Table S5). Total RNA (2 μg) from the samples was used for cDNA synthesis by M-MLV reverse transcriptase (Promega) with random primers (Takara). The expression levels of genes were analyzed using a MyGo Pro Real-Time PCR System (IT-IS Life Science, UK). The amplification of the *ELF1B* gene was used as a reference to normalize the expression levels. In the RT-qPCR experiments, three biological replicates were used for analyses.

### Protein extraction

A total of 1–2 g plant tissues were grounded into powder in liquid nitrogen using mortar and pestle, and then transferred into a 50 mL centrifuge tube. Proteins were extracted as described by Komatsu^51^. The proteins were air-dried and re-suspended in lysis buffer (8 M urea, 5% CHAPS, 2 M thiourea and 2 mM tributylphosphine). The suspension was centrifuged at 20,000 g for 20 min at 25 °C, and the resulting supernatant was collected as the crude protein extract.

### Protein purification and digestion

Proteins (150 μg) were purified by phase separation in the organic layer. The volume was adjusted to 150 μL. A total of 600 μL methanol was added to the protein solution, which was thoroughly mixed before 150 μL chloroform and 400 μL water were added. The mixture was vortexed and centrifuged at 20,000 g for 5 min. The supernatant was discarded. A total of 400 μL methanol was added to the organic phase, and the samples were centrifuged at 20,000 g for 5 min. The pellets were dried and re-suspended in 50 mM NH_4_HCO_3_, reduced with 50 mM dithiothreitol for 30 min at 56 °C, and alkylated with 50 mM iodoacetamide for 30 min at 37 °C in darkness.

Trypsin Gold (Promega, Madison, WI, USA) was used to digest the proteins with the ratio of protein: trypsin of 40: 1 at 37 °C overnight. After trypsin digestion, the peptides were desalted with a Strata X C18 column (Phenomenex) and vacuum-dried according to the manufacturer’s protocol.

### Mass spectrometry detection

Data acquisition was performed with a TripleTOF 5600 System (SCIEX, Framingham, MA, USA) equipped with a Nanospray III source (SCIEX, Framingham, MA, USA), a pulled quartz tip as the emitter (New Objectives, Woburn, MA) and controlled with the software Analyst 1.6 (AB SCIEX, Concord, ON).

### Protein quantification

IQuant^52^ was used for quantitative analysis of the labeled peptides with isobaric tags. To assess the confidence of peptides, the PSMs were prefiltered at a PSM-level FDR of 1%. Protein FDR at 1% was based on the picked protein FDR strategy to control the rate of false-positive at the protein level^53^. The protein quantification process included the following steps, protein identification, tag impurity correction, data normalization, missing value imputation, protein ratio calculation, statistical analysis, and result presentation.

### Protein GO and KEGG pathway enrichment

We defined DEPs to be significantly regulated if the *P* value was less than 0.05. In GO enrichment analysis, we used the hyper geometric test to get the target GO terms. The principle of the KEGG pathway enrichment analysis of differentially expressed proteins was similar.

### GO and KEGG enrichment analyses based on transcriptome and proteome

Because the accumulation of proteins fell behind the expressions of genes, only the samples under the 24-h submergence treatment were taken for the proteome sequencing analysis. We performed the gene ontology (GO) enrichment analysis by transcriptome and proteome analyses at 24 h under submergence stress. Then, the enriched GO terms at 24 h were integrated with the transcriptome data from the intersection of four time points. The analysis of the KEGG pathways was the same as that of the GO terms.

### Analysis of the lignin content

The submergence treatment of Qihuang 34 was the same as above, and the sampling time points included 24, 96 and 192 h. For the control, the plants were untreated with submergence. The root of the seedlings was collected from the control and the submergence treatment groups, grinding in liquid nitrogen and stored in a drying oven until the weight no longer changed. Three biological replicates were performed for each sample, which contained 10 roots from 10 independent plants. The lignin content was measured using a lignin extraction kit (COMIN, http://www.cominbio.com/).

## Supplementary information

Supplementary infomation

Table S2

Table S3

## Data Availability

The raw RNA-seq data were deposited in the NCBI Sequence Read Archive (SRA), and the Accession Number is SRP181976.
