# Supplementary material for: Identification of Genes/Proteins Related to Submergence Tolerance by Transcriptome and Proteome Analyses in Soybean
Source: Sci Rep. 2019 Oct 11;9:14688. doi: 10.1038/s41598-019-50757-1 (PMC6789146; doi:10.1038/s41598-019-50757-1)
Supplement: Supplementary file 1 — Supplementary infomation [file 41598_2019_50757_MOESM1_ESM.docx]

**Title：**Identification of Genes/Proteins Related to Submergence Tolerance by Transcriptome and Proteome Analyses in Soybean

**Author names and affiliations:**

***Yanhui Lin***

Crop Research Institute, Shandong Academy of Agricultural Sciences, Jinan, China

Institute of Food Crops, Hainan Academy of Agricultural Sciences, Haikou, China

E-mail: [lyh_1012@163.com](mailto:lyh_1012@163.com)

***Wei Li***

Crop Research Institute, Shandong Academy of Agricultural Sciences, Jinan, China

E-mail: [liwwqling@163.com](mailto:liwwqling@163.com)

***Yanwei Zhang***

Crop Research Institute, Shandong Academy of Agricultural Sciences, Jinan, China

E-mail: [zywei-1987@163.com](mailto:zywei-1987@163.com)

***Changjian Xia***

Haikou Cigar Research Institute, Hainan Provincial Branch of China National Tobacco Corporation, Haikou, China

State Key Laboratory for Biology of Plant Diseases and Insect Pests, Institute of Plant Protection, Chinese Academy of Agricultural Sciences, Beijing, China

E-mail: jeffreyshya@163.com

***Yun Liu***

College of Agriculture, Yangtze University, Jingzhou, China

E-mail: LY874976064@163.com

***Caijie Wang***

Crop Research Institute, Shandong Academy of Agricultural Sciences, Jinan, China

E-mail: [beautifulljie@sina.com](mailto:beautifulljie@sina.com)

***Ran Xu***

Crop Research Institute, Shandong Academy of Agricultural Sciences, Jinan, China

E-mail: soybeanxu@126.com

**Corresponding author:**

***Lifeng Zhang****

Crop Research Institute，Shandong Academy of Agricultural Sciences，Jinan，China

E-mail: zlfsoybean@163.com


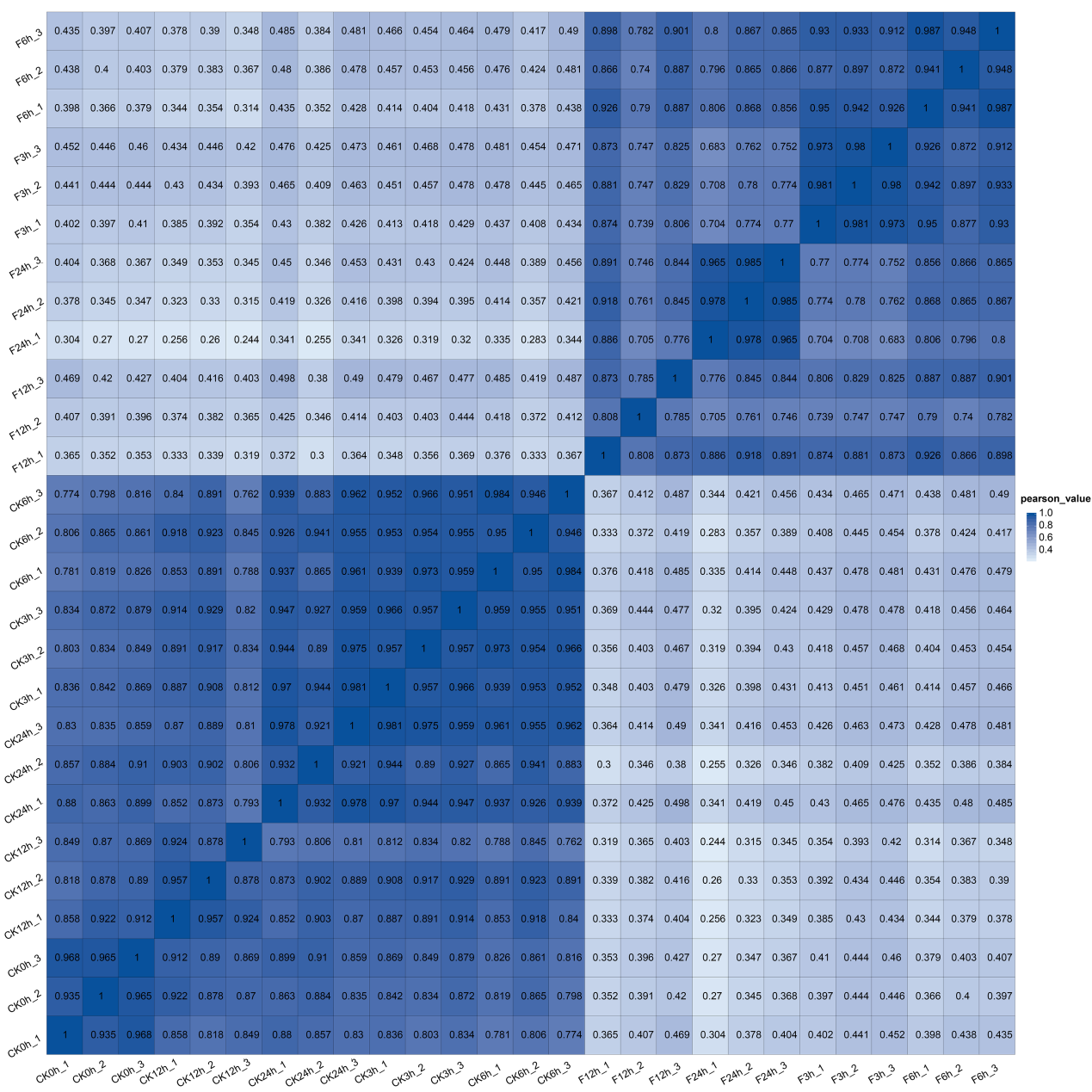


Fig. S1 Pearson correlation analysis between samples at 3, 6, 12 and 24 h. The R2 value close to 1 indicates a high correlation between samples. Each square represents the correlation of the two samples, and the darker color indicates a higher correlation. CK: Control; F: Flooding (submergence treatment).


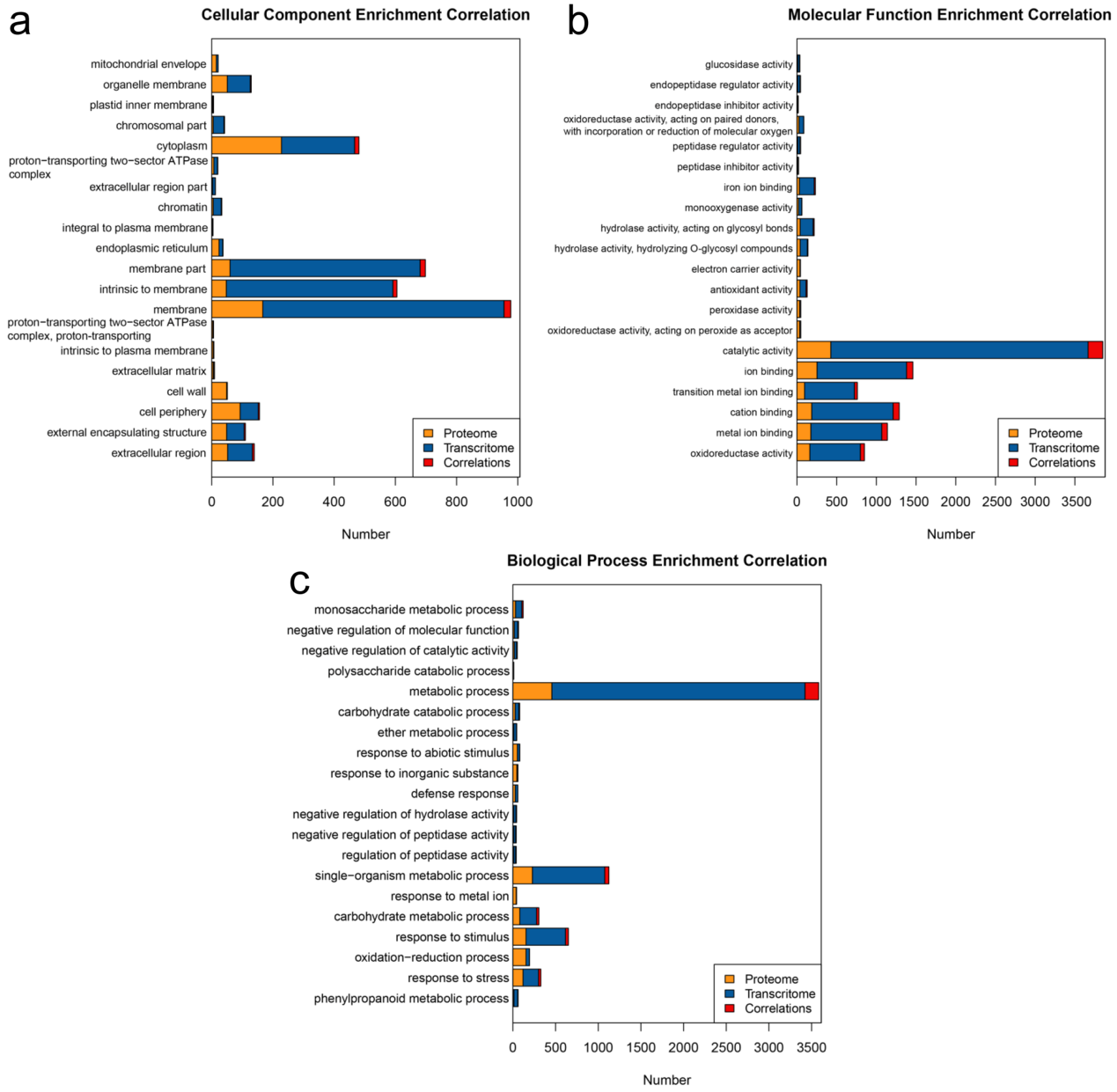


Fig. S2 GO enrichment analysis at the 24 h time point, showing the numbers of genes, functional proteins, and associated genes and functional proteins. Blue, transcriptome data; yellow, proteome data; red, correlation data.


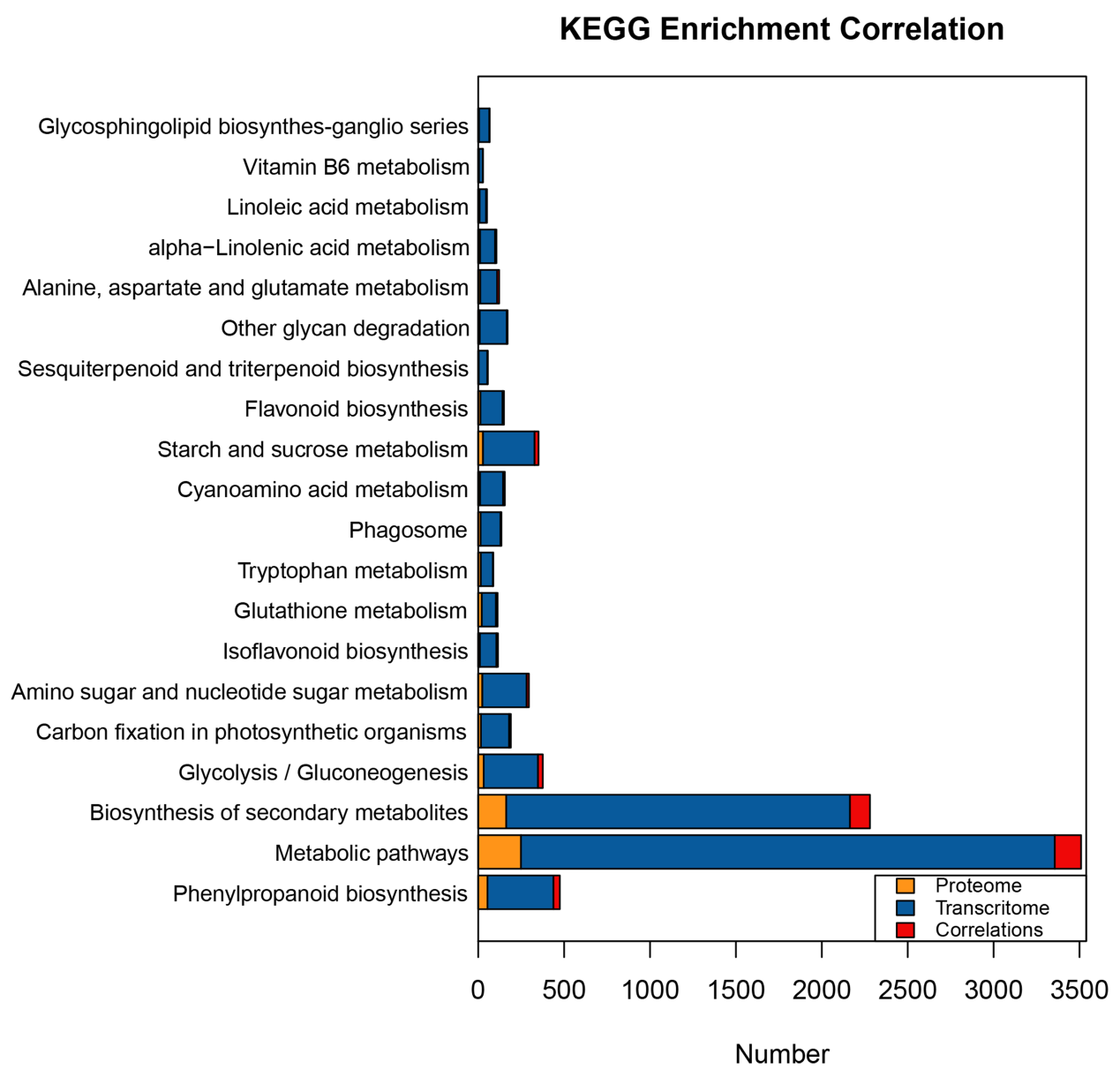


Fig. S3 KEGG pathway enrichment analysis at the 24 h time point, showing the numbers of genes, functional proteins, and associated genes and functional proteins. Blue, transcriptome data; yellow, proteome data; red, correlations data.


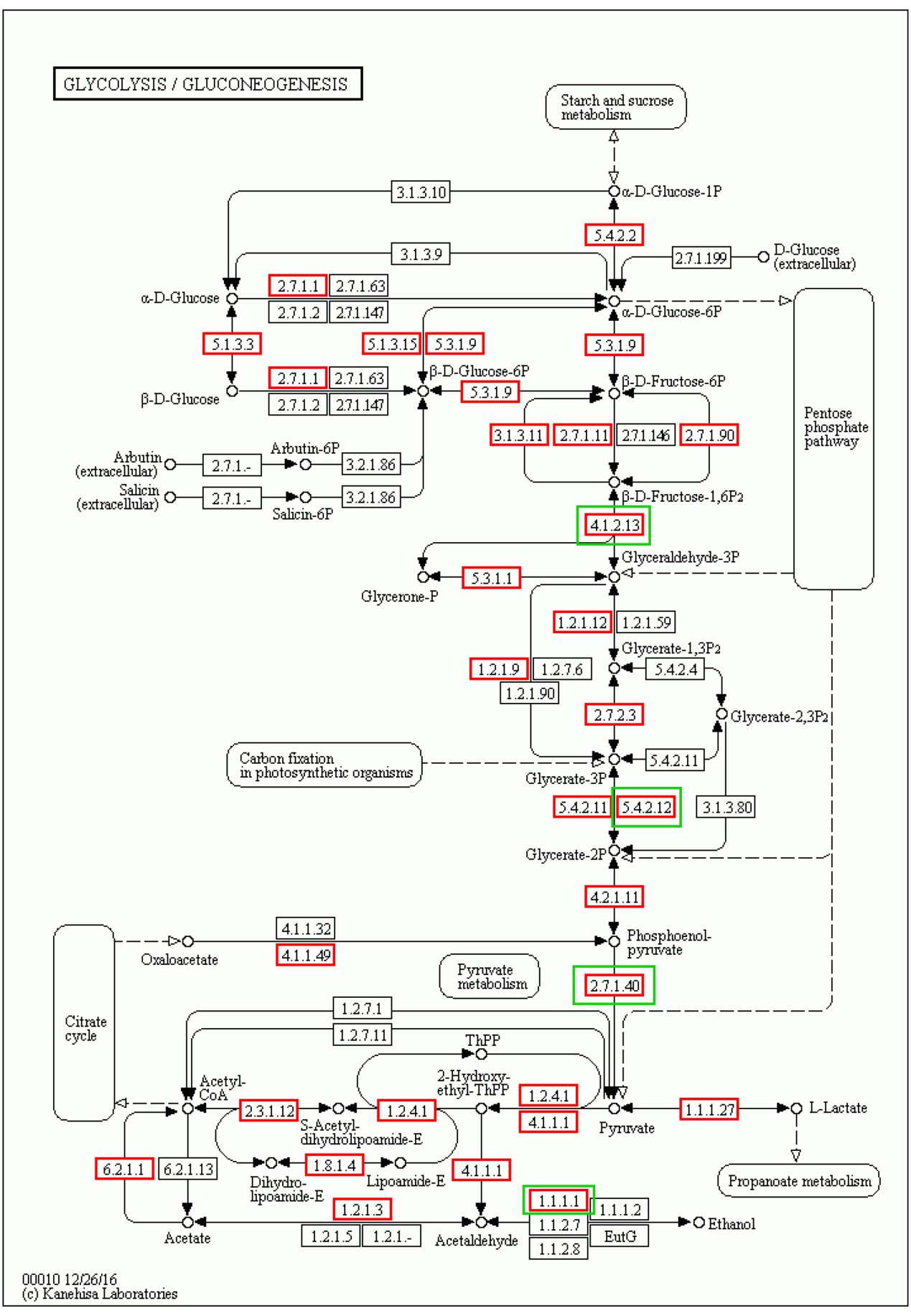


Fig. S4 DEGs/DEPs predicted to be involved in the glycolysis/gluconeogenesis pathway derived from KEGG database^53^. Green box indicate that the locations of the key enzyme-encoding genes participating in the glycolysis/gluconeogenesis.


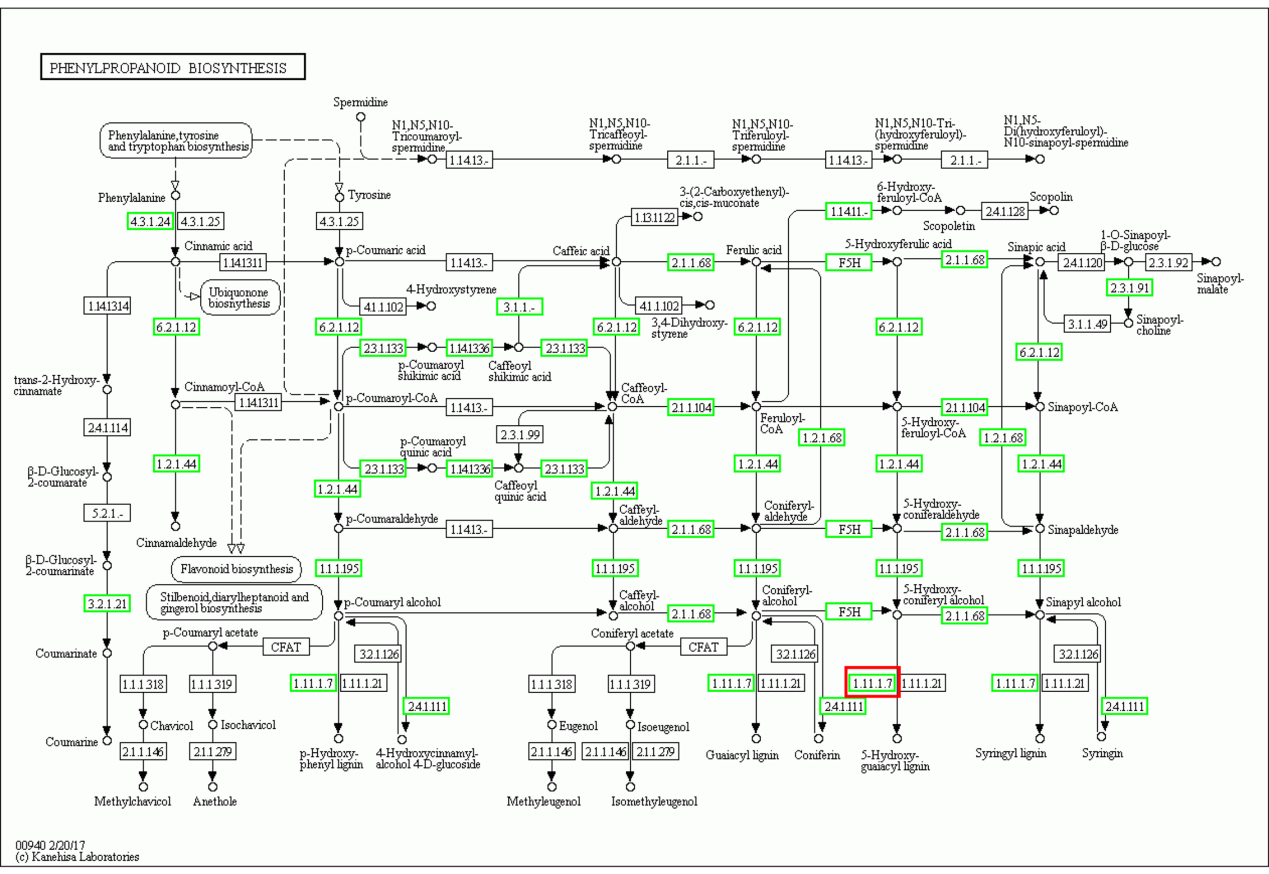


Fig. S5 DEGs/DEPs predicted to be involved in the phenylpropanoid biosynthesis pathway derived from KEGG database^53^. Red box indicates that the selected eight genes catalyze the conversion of coumaryl alcohol to hydroxy-phenyl lignin.


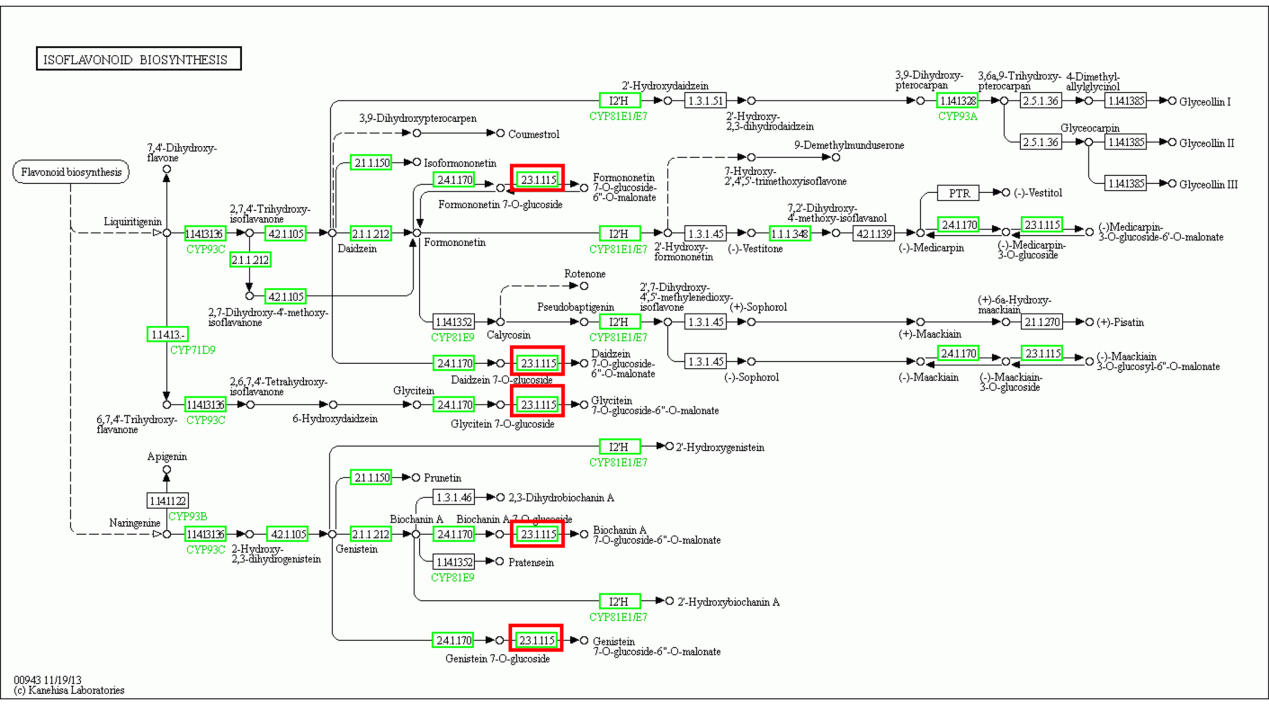


Fig. S6 DEG/DEP predicted to be involved in the isoflavonoid biosynthesis pathway derived from KEGG database^53^. Red box indicate that *Glyma.18G258000* catalyze the transformation of (daidzein, glycitein, biochanin and genistein) 7-O-glucoside to (daidzein, glycitein, biochanin and genistein) 7-O-glucoside-6''-malonate.


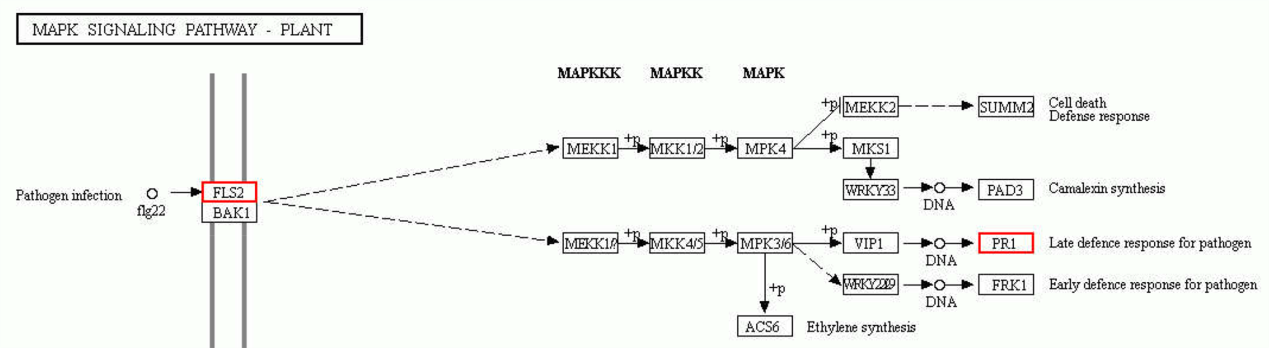


Fig. S7 DEGs/DEPs predicted to be involved in the MAPK signaling pathway derived from KEGG database^53^. FLS2 is a key signal transduction protein，which is encoded by three genes involved in MAPK signaling pathway-plant.

**Table S1** Summary of RNA-Seq performed for soybean root tissue under submergence stress.

| **Sample** | **Raw**  **Reads** | **Clean Reads** | **Clean Bases(G)** | **Q20 (%)** | **Q30 (%)** | **Clean Reads Ratio (%)** | **Total Mapping Ratio** |
| --- | --- | --- | --- | --- | --- | --- | --- |
| CK0h_1 | 23353882 | 23202082 | 1.16 | 98.83 | 93.23 | 99.35 | 89.33 |
| CK0h_2 | 23346103 | 23194353 | 1.16 | 98.90 | 93.43 | 99.53 | 88.77 |
| CK0h_3 | 23404982 | 23252850 | 1.16 | 98.94 | 93.73 | 99.07 | 89.43 |
| CK3h_1 | 23519365 | 23366489 | 1.17 | 98.71 | 92.48 | 99.73 | 91.11 |
| CK3h_2 | 23622564 | 23469017 | 1.17 | 98.80 | 92.96 | 99.88 | 90.88 |
| CK3h_3 | 23332078 | 23180419 | 1.16 | 98.85 | 93.15 | 99.33 | 90.59 |
| CK6h_1 | 23457494 | 23305020 | 1.17 | 98.76 | 92.83 | 99.67 | 91.60 |
| CK6h_2 | 23812445 | 23657664 | 1.18 | 98.83 | 93.07 | 99.75 | 90.95 |
| CK6h_3 | 23535059 | 23382081 | 1.17 | 98.80 | 92.86 | 99.86 | 91.37 |
| CK12h_1 | 23351002 | 23199220 | 1.16 | 98.92 | 93.58 | 99.47 | 91.60 |
| CK12h_2 | 23609488 | 23456026 | 1.17 | 98.90 | 93.24 | 99.75 | 90.95 |
| CK12h_3 | 23403044 | 23250924 | 1.16 | 98.88 | 93.54 | 99.01 | 91.37 |
| CK24h_1 | 23514904 | 23362057 | 1.17 | 98.80 | 92.89 | 99.47 | 91.26 |
| CK24h_2 | 23521999 | 23369106 | 1.17 | 98.86 | 93.18 | 99.84 | 89.73 |
| CK24h_3 | 23599575 | 23446178 | 1.17 | 98.67 | 92.15 | 99.90 | 91.55 |
| F3h_1 | 23830770 | 23675870 | 1.18 | 98.36 | 91.86 | 98.84 | 91.21 |
| F3h_2 | 23822587 | 23667740 | 1.18 | 98.36 | 91.83 | 98.84 | 90.78 |
| F3h_3 | 23649015 | 23495296 | 1.17 | 98.38 | 91.94 | 98.10 | 91.02 |
| F6h_1 | 23941552 | 23785932 | 1.19 | 98.24 | 91.41 | 99.37 | 91.49 |
| F6h_2 | 23671491 | 23517626 | 1.18 | 98.25 | 91.50 | 98.36 | 91.39 |
| F6h_3 | 23853117 | 23698072 | 1.18 | 98.33 | 91.71 | 98.86 | 91.10 |
| F12h_1 | 23456241 | 23303775 | 1.17 | 98.50 | 92.30 | 97.17 | 90.13 |
| F12h_2 | 23917278 | 23761816 | 1.19 | 98.34 | 91.26 | 98.45 | 86.65 |
| F12h_3 | 23694800 | 23540784 | 1.18 | 98.37 | 91.94 | 98.32 | 89.03 |
| F24h_1 | 23824307 | 23669449 | 1.18 | 98.15 | 91.07 | 98.96 | 92.07 |
| F24h_2 | 23853612 | 23698564 | 1.18 | 98.25 | 91.41 | 99.11 | 91.59 |
| F24h_3 | 23821484 | 23666644 | 1.18 | 98.22 | 91.31 | 98.97 | 92.07 |

**Table S2** The detail information of DEGs in all the sample combinations.

The data was showed in Excel file as Supplementary dataset.

**Table S3** The protein data under the 24 h submergence treatment.

The data was showed in Excel file as Supplementary dataset.

**Table S4** The same proteins in our study compared with those of the previously reported studies.

**Table S5** Primers used for RT-qPCR.

| **Gene/ Protein ID/**  **Accession Number** | **Gene/Protein in our study** | **Transcriptome/Proteome** | **reference** |
| --- | --- | --- | --- |
| Glyma15g41550(a1v1) | Glyma.15g262100(a2v1) | Proteome | Oh et al. 2015 |
| Glyma08g17600 | Glyma.08g165400 | Proteome | Oh et al. 2015 |
| Glyma18g52250 | Glyma.18g285800 | Proteome | Oh et al. 2015 |
| Glyma08g07950 | Glyma.08g074700 | Proteome | Oh et al. 2015 |
| CAI99393 | Glyma.05G123900 | Proteome | Komatsu et al. 2009 |
| CAA11075 | Glyma.08G200200 | Proteome | Komatsu et al. 2009; Hashiguchi et al.2009 |

| **Primer** | **Forward primer (5'-3')** | **Reverse primer (5'-3')** |
| --- | --- | --- |
| *ELF1B* | GTTGAAAAGCCAGGGGACA | TCTTACCCCTTGAGCGTGG |
| *Glyma.19G000700* | CAGGAAACCCTCAACAAT | GTCCCATCAGAGCACAAT |
| *Glyma.06G275900* | GACGGAATTGTGCTGCTA | TCCTGGGATTTGGTTGTT |
| *Glyma.06G176200* | AGCGACACCACTTCAACA | GGAATATCGTACCCTCTTA |
| *Glyma.16G204600* | TGGGAGCACTATGCGAAAT | AAGCCTTCTCCTTGATTG |
| *Glyma.04G213900* | TGACCGAAGTGTTGAGTG | AACGTGGTTTATAGTTGC |
| *Glyma.05G124000* | CAAACGCTACTCCAAATC | GTCGCATGAGACACCTAC |
| *Glyma.02G222400* | GGTCACTCTCTTTCTCCTTTGG | CTGAGTTAGCCTTGGCTCTTAC |
| *Glyma.14G053600* | CCAATGCCAATCCAACACTTC | ATGCGGGTCTCCTTTGATATG |
| *Glyma.18G219100* | GGGCATACAGGTGACATTGA | TTCCACCCACTTGCTCTATTG |
| *Glyma.03G039800* | TCAGATTTACCAGCTCCCTTTC | GCACCTGATAAAGCAACCATTT |
| *Glyma.18G258000* | TAGAGCCTCCAATCCCTGATAC | GACGAGCCCTTCTTCCTTTATG |
